# Microthrombotic Renal Vascular Lesions Are Associated to Increased Renal Inflammatory Infiltration in Murine Lupus Nephritis

**DOI:** 10.3389/fimmu.2018.01948

**Published:** 2018-08-28

**Authors:** Elena Gonzalo-Gil, Carmen García-Herrero, Oscar Toldos, Alicia Usategui, Gabriel Criado, Sonia Pérez-Yagüe, Domingo F. Barber, Jose L. Pablos, Maria Galindo

**Affiliations:** ^1^Instituto de Investigación, Hospital 12 de Octubre, Madrid, Spain; ^2^Servicio de Anatomía Patológica, Hospital Universitario 12 de Octubre, Madrid, Spain; ^3^Centro Nacional de Biotecnología (CNB-CSIC), Madrid, Spain; ^4^Servicio de Reumatología, Hospital Universitario 12 de Octubre, Madrid, Spain; ^5^Universidad Complutense de Madrid, Madrid, Spain

**Keywords:** microthrombosis, inflammation, lupus nephritis, complement, macrophages, platelets

## Abstract

**Background:** Vascular microthrombotic lesions in lupus nephritis with or without antiphospholipid antibodies may relate to worse renal outcomes. Whether microthrombotic lesions are a consequence of renal inflammation or independently contribute to renal damage is unclear. Our aim was to investigate the relationship between microthrombotic renal vascular lesions and nephritis progression in MRL/lpr mice.

**Methods:** MRL/lpr mice were analyzed for the presence of renal microvascular, glomerular and tubulointerstitial lesions and the effect of anti-aggregation (aspirin or clopidogrel) and dexamethasone on renal clinical and pathological manifestations was evaluated. Intravascular platelet aggregates (CD41), peri- (F4/80), and intraglomerular (Mac-2) macrophage infiltration, and C3 deposition were quantified by immunohistochemistry. Renal function was assessed by measuring proteinuria, and serum levels of creatinine and albumin. Anti-dsDNA and anti-cardiolipin antibodies, and thromboxane B2 levels were quantified by ELISA.

**Results:** Frequency of microthrombotic renal lesions in MRL/lpr mice was high and was associated with immune-mediated renal damage. Proteinuria positively correlated with glomerular macrophage infiltration and was higher in mice with proliferative glomerular lesions. All mice had detectable anti-dsDNA and anti-cardiolipin IgG, regardless the presence of microthrombosis. Proteinuria and glomerular macrophage infiltration were significantly reduced in all treatment groups. Dexamethasone and platelet anti-aggregation similarly reduced glomerular damage and inflammation, but only platelet anti-aggregation significantly reduced anti-cardiolipin antibodies, renal complement deposition and thromboxane B2 levels.

**Conclusions:** Platelet anti-aggregation reduced renal inflammatory damage, renal complement deposition, anti-cardiolipin antibodies, and thromboxane B2 levels and in MRL/lpr mice, suggesting that platelet activation has a pathogenic effect on immune-mediated nephritis. Our results point to MRL/lpr mice with lupus nephritis as an appropriate model to analyze the potential impact of anti-thrombotic intervention on renal inflammation.

## Introduction

Clinical evidence of lupus nephritis (LN) occurs in up to 50% of the patients with systemic lupus erythematosus (SLE) (1) and is a major cause of morbidity and mortality. In fact, renal injury is the most important predictor of mortality in patients with SLE (2). In addition to classical glomerulonephritis lesions, the presence of microvascular lesions may also adversely affect the course of renal disease (3, 4). Among renal vasculopathies, including vascular immune complex deposition, noninflammatory necrotizing vasculopathy, thrombotic microangiopathy (TMA), and true renal vasculitis, TMA presents the most severe clinical manifestations and highest mortality rate (3, 5–7). Non-specific and chronic atherosclerotic lesions can be found as a consequence of cardiovascular risk factors or previous acute vascular lesions. These vascular lesions have been reported particularly in patients with SLE and positive antiphospholipid antibodies (aPL), but also in their absence (8, 9).

The underlying mechanism for development of aPL-associated thrombosis is not fully understood and a question still unresolved is whether it is dependent on the coexistence of an inflammatory response. In 87.5% of patients, it has been described an association between platelet activation and SLE (10), a mechanism dependent of CD40-CD40L interaction and the presence of aPL (11). Additionally, the presence of aPL induces the expression of tissue factor (TF) on the surface of endothelial cells and peripheral blood monocytes (12, 13). In animal models of antiphospholipid syndrome (APS), activation of the complement system is necessary for the development of fetal loss and thrombosis (14, 15). Besides routine histological methods (16), immunohistochemistry (IHC) detection of platelet aggregates on kidney sections increases the sensitivity to detect acute microthrombosis (17, 18). However, whether microthrombotic lesions are a consequence of renal inflammation or independently contribute to renal damage is unclear (5, 16, 19). In patients with LN, intravascular CD61+ microthrombosis has been associated with higher glomerular infiltration by CD68+ macrophages (18), a validated activity and severity marker in proliferative LN. Whether microthrombotic lesions are the cause or the consequence of more severe LN is not known.

MRL/MpJ-*Fas*^*lpr*^/J (MRL/lpr) mice, with a genetic deficiency in Fas receptor, are characterized by lymphoproliferation and severe autoimmune disease that includes renal involvement (20). Here we use MRL/lpr mice with LN to study the relationship between nephritis progression and renal microthrombosis by testing the effect of anti-thrombotic therapies in the course of renal pathology.

## Materials and methods

### Mouse model

MRL/lpr mice were bred at Centro Nacional de Biotecnología animal facility and maintained under specific pathogen-free conditions. Mice were sacrificed under isoflurane anesthesia (Forane) and all efforts were made to avoid suffering. All animal experiments followed institutional guidelines and all protocols were approved by the ethics committees of Hospital 12 de Octubre and the CSIC (Centro Superior de Investigaciones Científicas).

### Renal involvement in MRL/lpr mice

#### Renal function measurements

Renal function was assessed by quantification of proteinuria, and levels of serum creatinine (sCr) and serum albumin (sAlb). The degree of proteinuria was estimated and quantified by using urine dipstick (Aution Sticks, Arkray Europe, BV, Amstelveen, the Netherlands). Blood was extracted by cardiac puncture at the time of sacrifice. Serum was obtained from whole blood by centrifugation at 2,000 g for 10 min at 4 degrees and stored at −20° for further use. Albumin and creatinine concentration in mice serum was analyzed using a chemistry basic colorimetric test. Data was evaluated in SPOTCHEM EZ Analyzer (SP-4430; Arkray Factory, Arkray Europe, BV, Amstelveen, The Netherlands).

#### Enzyme-linked immunosorbent assay

Level of anti-dsDNA and anti-Cardiolipin (aCL) IgG antibodies (Alpha diagnostic international; San Antonio, TX, USA), and thromboxane B2 (TxB2) (Elabscience Biotechnology, Wuhan, China) were quantified by ELISA following the manufacturer's recommendations. Concentration of the antibodies and thromboxane was calculated using the standard curve method.

#### Histological and immunohistochemistry analysis in renal tissue

Kidneys were extracted at the time of sacrifice and were transfused with physiological saline before being processed for histological studies. The pattern of renal vascular disease, acute and chronic glomerular and tubulointerstitial lesions were specifically analyzed by using hematoxylin-eosin (H&E), periodic acid-Schiff (PAS), and Masson's trichromic stains, in 4 microns paraffin-embedded kidney sections. Kidney histology was evaluated and classified according to parameters used in SLE patients (21) by a pathologist who was blinded to clinical and laboratory information. Percentage of glomerular sclerosis in at least 25% of analyzed glomeruli and degree of hypercellularity (from 0 to 3) were calculated. Tubulointerstitial injury degree was evaluated as follows: (0) absence of cellular component; (1) between 1 and 5 intratubular cellular components with minimal fibrosis; (2) between 5 and 10 cells with moderate fibrosis; (3) more than 10 cells with severe fibrosis.

**Figure 1:**
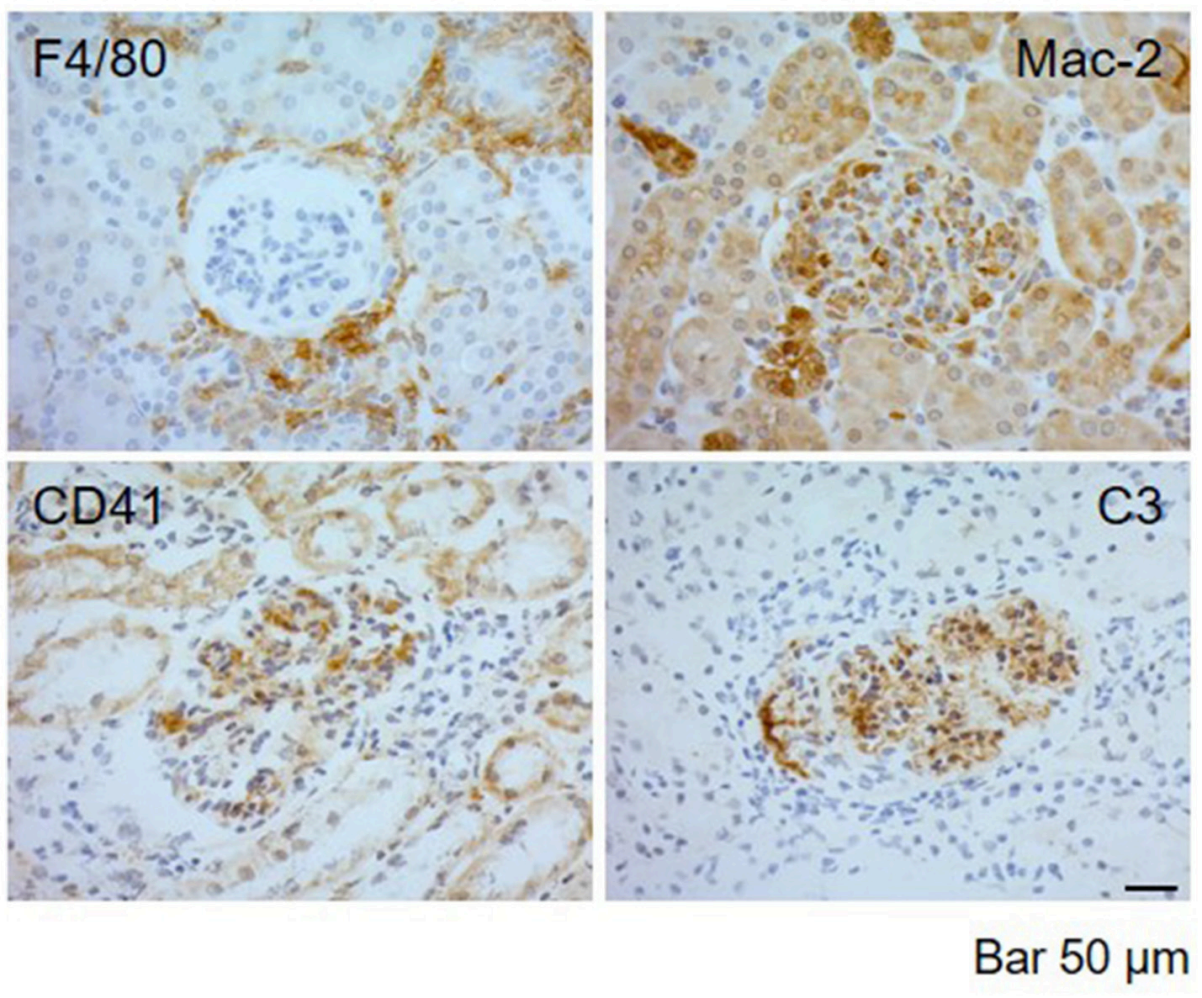
Representative images of immunohistochemical detection in kidney biopsies of MRL/ lpr mice with LN. Immunolabeling detection of F4/80 periglomerular macrophagic deposits, Mac-2 intraglomerular macrophagic infiltration, CD41+ platelet aggregates, and C3 complement deposition **i**n MRL/ lpr mice with LN. Bar 50 μm.

IHC techniques were used to detect periglomerular (F4/80, clone BM8; eBioscience; San Diego, CA, USA) and intraglomerular macrophage infiltration (Mac-2, clone M3/38; Biolegend; CA, USA), complement deposition (C3b/iC3b/C3c, clone 2/11; Hycult Biotech Deltaclon; Madrid, Spain), and mouse Integrin α IIb chain (CD41, clone MWReg30; BD Pharmigen; San Diego CA, USA). A representative staining is included in Figure 1. To detect F4/80 and MAC-2, sections were deparaffinized and pretreated with 10 μg/ml proteinase K (P6556; Sigma-Aldrich Química SA, Madrid, Spain) in the case of F4/80 detection, or boiled for 20 min in citrate buffer 10 mM, pH 6, for MAC-2 detection. C3 and CD41 were detected in frozen sections fixed with 4% PFA for 10 min. The slides were incubated overnight at 4°C with primary antibodies. IHC staining was performed with a peroxidase avidin-biotin complex technique (SK-4100; Vector Laboratories Inc., Burlingame, CA, USA). Diaminobenzidine was used as chromogen substrate, and sections were counterstained with hematoxylin. The percentage of glomeruli with periglomerular F4/80 macrophage infiltration was quantified. Mac-2 intraglomerular macrophages were quantified as the fractional immunostained area within all visible glomeruli and C3 as the fractional area of the total evaluated tissue area. C3 and Mac-2 intraglomerular staining areas were quantified by using Image J software [Image J: Image processing and analysis in Java. (http://rsb.info.nih.gov/ij).], and percentage of stained area in 10 glomeruli and 10 extraglomerular areas randomly selected from each sample was determined (see Figure 1).

Two independent observers analyzed all slides. CD41 detection was analyzed as presence or absence of platelet microthrombi aggregates. Additionally, we analyzed the presence of acute vascular lesions as thrombotic microangiopathy (TMA), characterized by the presence of fibrin thrombi in arterioles and/or glomeruli, and severe chronic lesions such as myofibroblastic intimal cellular proliferation leading to intimal thickening of interlobular arteries or fibrous intimal hyperplasia (FIH), organized thrombi with or without recanalization, fibrous arterial or arteriolar occlusion and subcapsular zone focal cortical atrophy (FCA) (16, 22, 23). Thus, the presence of microthrombosis was defined as the presence of TMA or CD41+ platelet aggregates.

### Treatment protocols

Fourteen-weeks-old female MRL/lpr mice were treated intraperitoneally (i.p.) every day for 2 weeks according to the following strategies: dexamethasone (Dexa, 15 mg/day) to inhibit inflammatory response, and aspirin (ASA, 10 mg/Kg/day) or clopidogrel (Clop, 1.5 mg/Kg/day) for anti-aggregation purposes according to previous published data (24, 25). The effect of the treatment in vascular lesions and inflammatory responses was compared with a control group (PBS i.p.). A total of 3 experiments were performed in the same conditions.

### Statistical analysis

A descriptive analysis was firstly performed. Association between categorical variables was tested by the chi-square test. For continuous variables, comparisons were carried out using U-Mann Whitney *t*-test for two independent samples or one-way ANOVA followed by Dunn's multiple comparison test. To analyze the correlation among different study parameters a Spearman's or Pearson's rank were used as needed. Analysis was performed using GraphPad Prism software (version 6.0, Graph Pad; CA, USA) and advanced SPSS software (version 15.0, SPSS Iberica, Madrid, Spain). *P*- values < 0.05 were considered significant.

## Results

### Renal involvement in MRL/lpr mice

First, we analyzed the renal affectation in female MRL/lpr mice at 12-weeks (*n* = 13), 16-weeks (*n* = 20), and 20-weeks-old (*n* = 8) to define the renal involvement and to evaluate the progression of the LN. Three independent experiments were performed to stablish the time where most of the mice had renal damage. All age groups showed renal involvement, without differences in the frequency of diffuse proliferative GN lesions [77% (12w), 55% (16w), and 62.5% (20w)]. Likewise, levels of proteinuria and serum creatinine were elevated but we did not find differences over time (data not shown). Both, IgG aCL and anti-dsDNA levels were high and increased proportionally with age (aCL [U/ml]: 1.81 ± 0.2, 2.76 ± 0.11 and 2.9 ± 0.12 at 12, 16, and 20 w, respectively; anti-dsDNA [U/ml]: 8.98 ± 1.41 × 10^5^, 2.75 ± 1.9 × 10^6^ and 9.86 ± 9.23 × 10^6^ at 12, 16, and 20 w, respectively). However, the increased mortality rate in the 20-weeks-old group precluded the collection of data from this age group.

Therefore, to define the renal involvement and to further examine the effect of anti-inflammatory and anti-aggregate agents in the MRL/lpr lupus nephritis we selected 16-weeks old mice.

### Effect of anti-inflammatory agents and platelet anti-aggregation in MRL/lpr mice with LN

To evaluate the effect of inflammation and platelet aggregation in vascular renal disease, mice were treated with Dexa (*n* = 21), and with two different anti-aggregating agents, ASA (*n* = 15) or Clop (*n* = 15), and clinical and histopathological features were compared with PBS control group (*n* = 19). The effects of specific treatments are detailed in Table 1 and **Figures 3, 4**.

**Table 1:** Effect of anti-inflammatory and anti-platelet treatments on histological, IHC and clinical characteristics of MRL/lpr mice with lupus nephritis.

	**PBS (*n* = 19)**	**Dexa (*n* = 21)**	**ASA (*n* = 15)**	**Clop (*n* = 15)**
Proliferative GN (II+III+IV) (%)	7/19 (36.84)	7/21 (33.33)	5/15 (33.33)	4/15 (26.66)
Severe proliferative GN (III+IV) (%)	6/19 (31.58)	2/21 (9.52)	4/15 (26.67)	4/15 (26.67)
% Sclerosing glomeruli (min/max “*n*”)	0.32 (0/3)	0.19 (0/2)	0.27 (0/4)	1 (0/7)
Interstitial fibrosis	0	0	0	0
Tubular atrophy	0	0	0	0
sCr (mg/dl)^#^	0.89 ± 0.09	0.78 ± 0.04	0.73 ± 0.04	0.67 ± 0.03^∧^
sAlb (g/dl)^#^	2.43 ± 0.1	2.53 ± 0.07	2.39 ± 0.12	2.34 ± 0.06
Proteinuria (mg)^#^	431.39 ± 91.16	79.50 ± 20.1^†^	97.33 ± 43.80^†^	83.33 ± 30.69^†^
IgG anti-dsDNA (U/ml)^#^	905951.20 ± 98929.18	709348.03 ± 84443.87	757683.40 ± 95527.94	812042.40 ± 151174.30
IgG aCL (U/ml)^#^	15.52 ± 5.54	9.05 ± 2.49	9.65 ± 2.77	1.44 ± 0.13^∧^
Microthrombotic vascular lesions (%)^*^	13/19 (68.42)	10/21 (47.62)	6/15 (40)	9/15 (60)
Chronic vascular lesions (%)^**^	2/19 (10.53)	0	1/15 (6.67)	0
% C3 deposition^#^	6.56 ± 1.24	7.20 ± 1.37	3.52 ± 0.82^∧^	2.51 ± 0.95^∧^
% Mac-2^#^	5.88 ± 0.88	3.48 ± 0.35^∧^	4.23 ± 0.56	3.23 ± 0.45^∧^
% F4/80^#^	61.05 ± 4.12	38.36 ± 5.31^†^	43.46 ± 5.69^∧^	40.02 ± 4.15^†^
TxB2 (pg/ml)^#^	213.94 ± 24.52	239.10 ± 13.93	169.65 ± 38.05	114.42 ± 17.93^†^

*n, number; GN, glomerulonephritis; sCr, serum creatinine; sAlb, serum albumin; aCL, anti-cardiolipin antibodies; C3, complement deposition; Mac-2, intraglomerular macrophages; F4/80, periglomerular macrophage infiltration; TxB2, thromboxane B2 levels*.

# Data presented as mean ± SEM;

* *Microthrombotic vascular lesions, presence of thrombotic microangiopathy (TMA) or CD41+ platelet aggregates*,

** Chronic vascular lesions: presence of thyroidization, atherosclerosis, intimal fibrotic hyperplasia, organized thrombi, arteriolar hyalinosis or sclerosis, vascular occlusions or focal cortical atrophy;

∧ *p ≤ 0.05, statistical differences compared to PBS control group*,

† *p ≤ 0.01, statistical differences compared to PBS control group. Pooled data from three experiments performed in the same conditions*.

**Figure 2:**
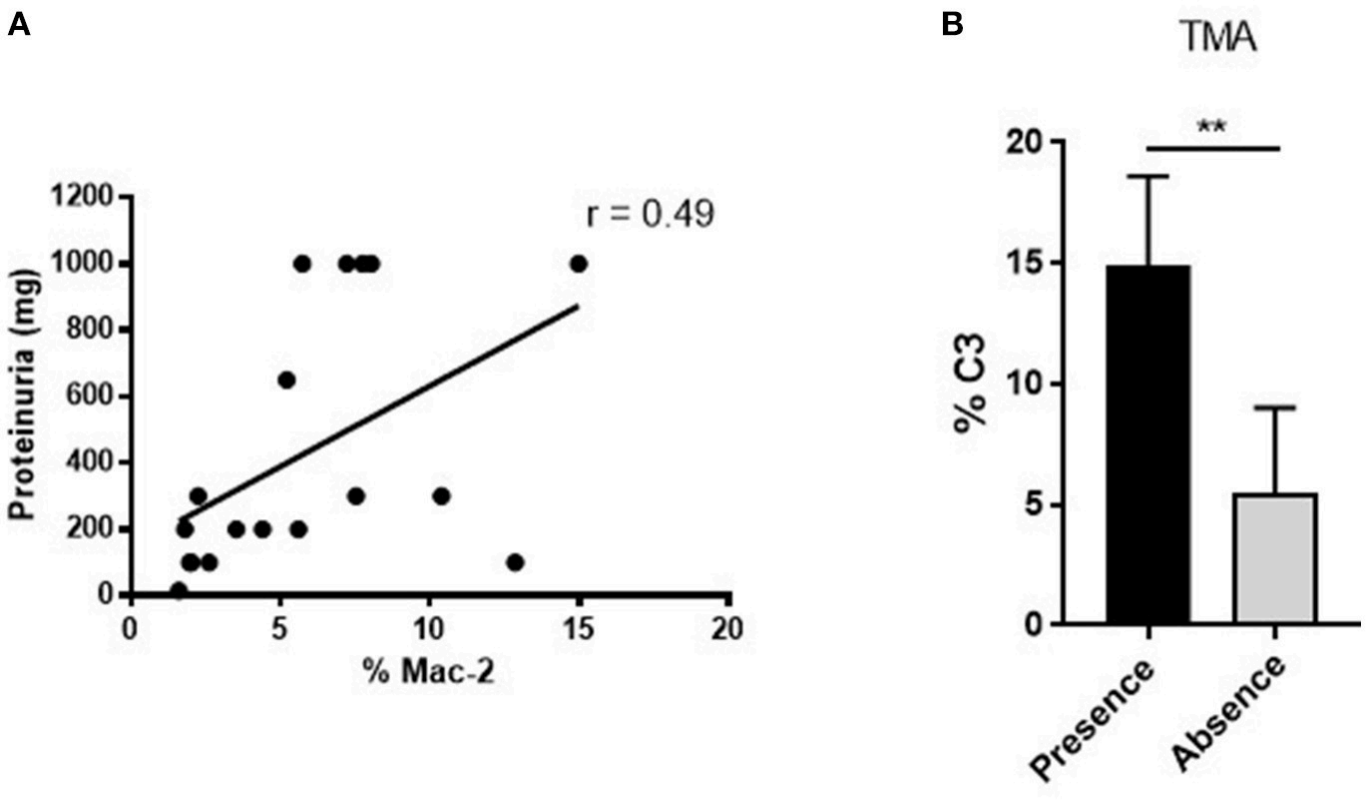
Positive associations between renal function measurement, vascular and immunohistochemical parameters in MRL/ lpr mice with LN. **(A)** Positive correlation between Mac-2 glomerular infiltration and degree of proteinuria in MRL/lpr mice with LN. Values obtained using the parametric Pearson test, ^**^p ≤ 0.01. **(B)** Increased % of glomerular C3 deposition in mice with presence of microthrombotic lesions, measured as TMA, ^**^p ≤ 0.01.

One third (31.58%) of MRL/lpr mice developed severe proliferative GN in the PBS control group (Table 1, PBS group). Microthrombotic vascular lesions, defined by the presence of histological TMA or intravascular CD41+ platelet aggregates, were present in 68.42% of mice. Other chronic vascular lesions were present in 10.53% of them (Table 1, PBS group). Table 2 shows histological, IHC and clinical characteristics of MRL/lpr with lupus nephritis mice from control group (PBS). Mice with proliferative GN (types III + IV) showed a non-significant trend toward higher levels of proteinuria, sCr, anti-dsDNA antibodies, and aCL antibodies (Table 2, top panel). Intra- and periglomerular macrophagic infiltration was significantly higher in mice with proliferative GN lesions (*p* = 0.001 and *p* = 0.04, respectively; Table 2, top panel). Also, we confirmed that Mac-2 glomerular infiltration correlated positively with the degree of proteinuria (*r* = 0.49; *p* = 0.006; Figure 2A). Mice with microthrombotic vascular lesions showed a higher degree of proteinuria (*p* = 0.007; Table 2, bottom panel), and specifically, mice with histologic TMA had higher levels of IgG aCL antibodies (*p* = 0.02; Table 2, bottom panel) and higher degree of C3 deposition (*p* = 0.001, Figure 2B). No association was found between the presence of severe proliferative GN and microthrombotic vascular lesions (data not shown). These data demonstrate a high prevalence of microthrombotic lesions in LN of MRL/lpr mice and suggest an association between these lesions and immune-mediated renal damage.

**Table 2:** Histological, IHC and clinical characteristics of MRL/lpr with lupus nephritis mice from control group (PBS).

	**GN types III and IV (+) (*n* = 6)**	**GN types III and IV (–) (*n* = 13)**	***p*-value**
Proteinuria (mg)^#^	600 ± 180.74	347 ± 99.51	0.2
sCr (mg/dl)^#^	1 ± 0.25	0.85 ± 0.06	0.42
IgG anti-dsDNA (U/ml)^#^	11.04 × 10^5^ ± 1.48 × 10^5^	8.15 × 10^5^ ± 1.23 × 10^5^	0.18
IgG aCL (U/ml)^#^	27.08 ± 13.46	10.19 ± 5.0	0.16
Microthrombotic vascular lesions (%)^*^	66.67%	69.23	1
Chronic vascular lesions (%)^**^	33.34%	0%	0.09
% C3 deposition^#^	9.19 ± 2.72	5.35 ± 1.25	0.15
% Mac-2^#^	9.79 ± 1.47	4.08 ± 0.64	0.001
% F4/80^#^	70.39 ± 2.11	56.74 ± 5.61	0.04
	**Microthrombotic vascular lesions (**+**) (*****n*** = **13)**	**Microthrombotic vascular lesions (–) (*****n*** = **6)**	***p*****-value**
Proteinuria (mg)^#^	535.77 ± 113.82	160 ± 24.5	0.007
sCr (mg/dl)^#^	0.89 ± 0.12	0.9 ± 0.08	0.96
IgG anti-dsDNA (U/ml)^#^	9.63 × 10^5^ ± 1.05 × 10^5^	7.83 × 10^5^ ± 2.22 × 10^5^	0.41
IgG aCL (U/ml)^#^	13.93 ± 6.77 (TMA 43.98 ± 22.83)^∧^	18.98 ± 10.43 (TMA 10.19 ± 4.33)^∧^	0.68 (0.02)^∧^
% C3 deposition^#^	7.64 ± 1.49 (TMA 14.92 ± 2.13)^∧^	4.22 ± 2.09 (TMA 4.99 ± 1.02)^∧^	0.21 (0.001)^∧^
% Mac-2^#^	6.01 ± 1.08	5.61 ± 1.61	0.84
% F4/80^#^	59.25 ± 4.08	64.95 ± 10.14	0.54

*n, number; GN, glomerulonephritis; sCr, serum creatinine; aCL, anti-cardiolipin antibodies; C3, complement deposition; Mac-2, intraglomerular macrophages; F4/80, periglomerular macrophage infiltration*.

# Data presented as mean ±SEM;

* *Microthrombotic vascular lesions, presence of thrombotic microangiopathy (TMA) or CD41+ platelet aggregates*,

** *Chronic vascular lesions, presence of thyroidization, atherosclerosis, intimal fibrotic hyperplasia, organized thrombi, arteriolar hyalinosis or sclerosis, vascular occlusions or focal cortical atrophy*.

∧ *Microthrombotic vascular lesions considered as TMA alone. Pooled data from three experiments performed in the same conditions*.

**Figure 3:**
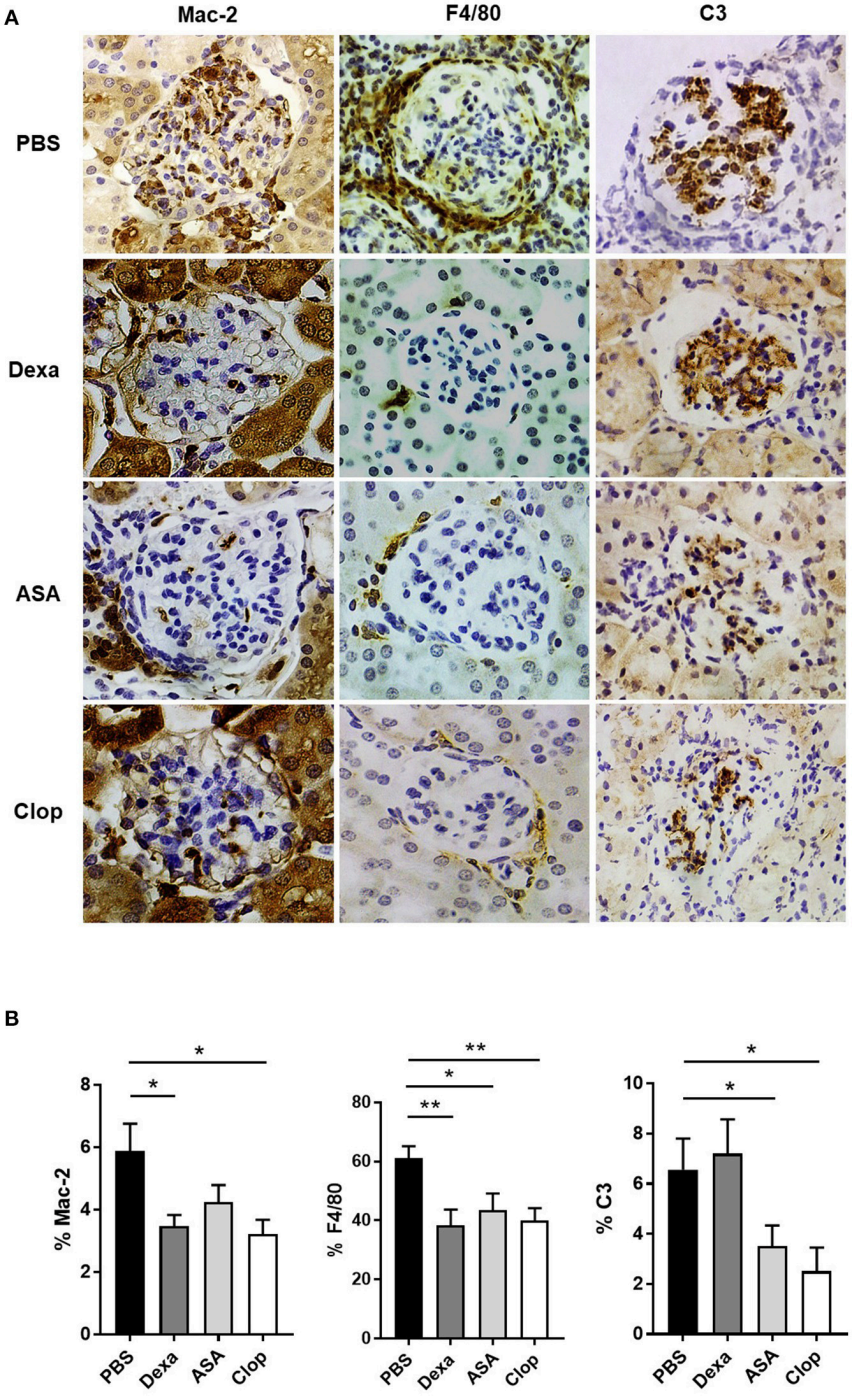
Effect of anti-inflammatories and anti-aggregants in MRL/ lpr mice with LN**. (A)** IHQ staining of Mac-2 (left), F4/80 (center), and C3 (right) in MRL/ lpr mice treated with Dexamethasone (Dexa), Aspirin (ASA), and Clopidogrel (Clop), compared to control group mice (PBS). **(B)** Anti-inflammatory and anti-aggregant effect in MRL/ lpr with LN in glomerular and periglomerular macrophagic deposits, determined as percentage of Mac-2 and F4/80, respectively, and glomerular complement deposition quantified as percentage of C3 area. Statistical analysis performed using U-Mann Whitney *t*-test for two independent samples or one-way ANOVA followed by Dunn's multiple comparison test, as needed. ^*^p ≤ 0.05; ^**^p ≤ 0.01.

**Figure 4:**
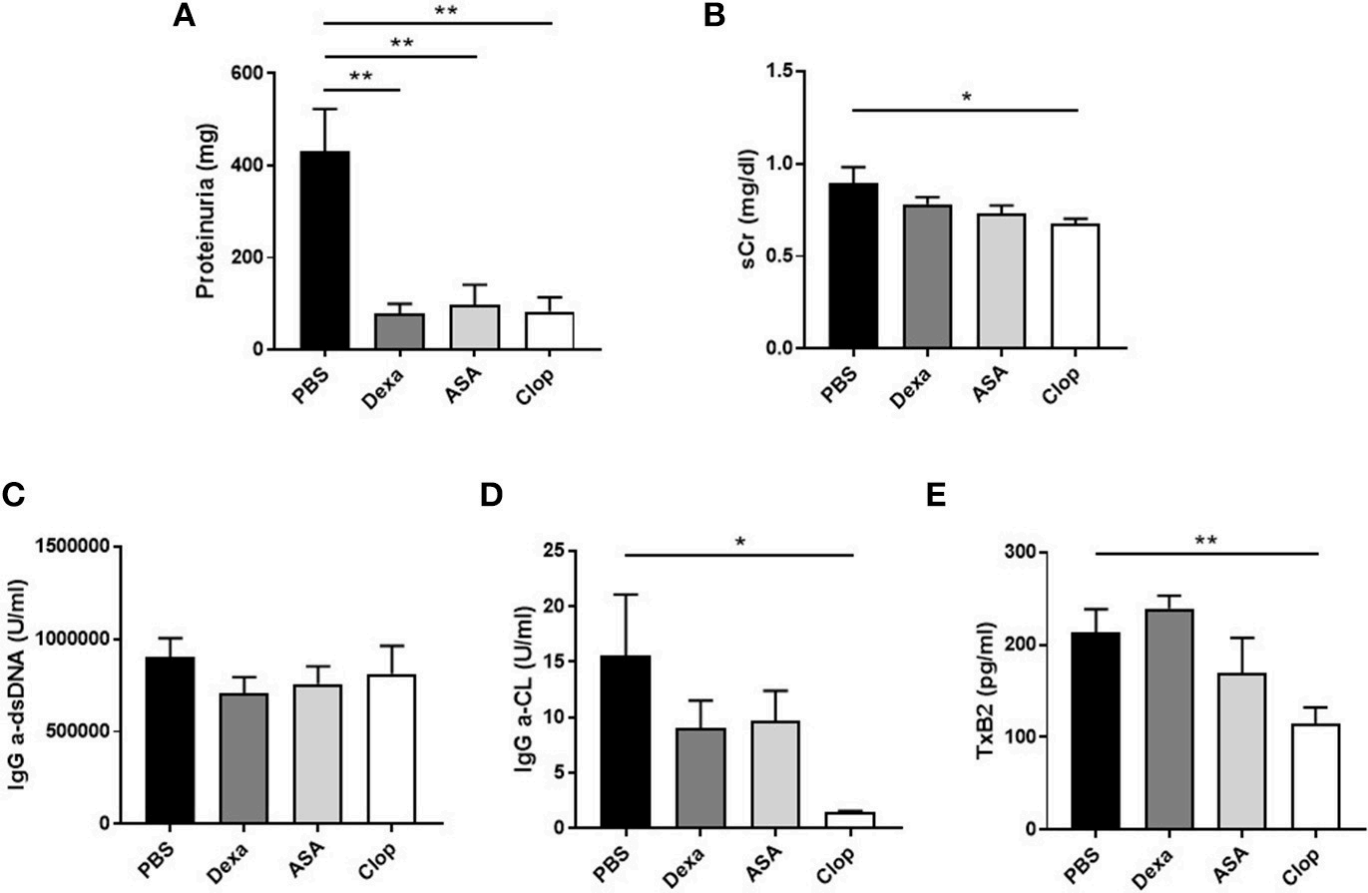
Effect of treatment with anti-inflammatory and anti-aggregation agents in proteinuria and serum parameters in MRL/ lpr mice. **(A)** Levels of proteinuria (mg) in MRL/ lpr mice. Serum creatinin (mg/dl) levels **(B)**, IgG anti-dsDNA (U/ml), and IgG anti-cardiolipin (U/ml) antibodies **(C–D)**, and serum levels of TxB2 (pg/ml) **(E)** in MRL/ lpr mice. Statistical analysis performed using one-way ANOVA followed by Dunn's multiple comparison test, as needed. ^*^p ≤ 0.05; ^**^p ≤ 0.01.

Mice treated with Dexa showed a non-significant trend toward reduced incidence of severe proliferative GN (PBS 31.58% vs. Dexa 9.52%) and of microthrombotic (PBS 68.42% vs. Dexa 47.62%), and chronic (PBS 10.53% vs. Dexa 0%) vascular lesions Table 1. Compared with the control group, treatment with Dexa significantly decreased the degree of glomerular (PBS 5.88 ± 0.88 vs. Dexa 3.48 ± 0.35, *p* = 0.018) and extraglomerular (PBS 61.05 ± 4.12 vs. Dexa 38.36 ± 5.31, *p* = 0.002) macrophage infiltration (Figures 3A,B) and reduced proteinuria (PBS 431.39 ± 91.16 vs. Dexa 79.50 ± 20.10 mg, *p* = 0.001; Figure 4A). No differences were found in C3 deposition (Figures 3A,B), or in the level of sCr and sAlb (Table 1). Treatment with Dexa did not affect the level of anti-dsDNA and aCL antibodies.

ASA anti-aggregation therapy significantly decreased proteinuria (PBS 431.39 ± 91.16 vs. ASA 97.33 ± 43.80; *p* = 0.001, Figure 4A), and extraglomerular macrophage infiltration (PBS 61.05 ± 4.12 vs. ASA 43.46 ± 5.69, *p* = 0.02, Figures 3A,B). Also, anti-aggregation using ASA significantly reduced C3 deposition (PBS 6.56 ± 1.24 vs. ASA 3.52 ± 0.82, *p* = 0.02; Figures 3A,B), pointing that anti-aggregation treatment has an effect in the inflammatory response.

Given the beneficial effect of ASA on the renal inflammatory response, and in order to confirm that the effect was due to blockade of platelet activation and not a direct anti-inflammatory effect of ASA, we performed the same experiments using Clop as antiaggregant agent. Treatment with Clop significantly reduced C3 deposition (PBS 6.56 ± 1.24 vs. Clop 2.51 ± 0.95; *p* = 0.017, Figures 3A,B), and decreased percentage of both intra- (PBS 5.88 ± 0.88 vs. Clop 3.23 ± 0.45; *p* = 0.02) and peri-glomerular macrophage infiltration (PBS 61.05 ± 4.12 vs. Clop 40.02 ± 4.15; *p* = 0.001, Figures 3A,B). Interestingly, Clop significantly reduced sCr and aCL antibodies levels (sCr: 0.89 ± 0.09 vs. 0.67 ± 0.03 mg/dl, *p* = 0.03; and aCL: 15.52 ± 5.54 vs. 1.44 ± 0.13 U/ml, *p* = 0.02, Figures 4B,D, respectively). Although ASA treatment also reduced the level of aCL antibodies, differences did not reach the statistical significance (Figure 4D).

Mice treated with ASA and Clop showed a non-significant reduction in the incidence of severe proliferative GN (PBS 31.58%; ASA or Clop 26.67%) and of microthrombotic (PBS 68.42%; ASA 40%; Clop 60%), and chronic (PBS 10.53%; ASA 6.67%; Clop0%) vascular lesions compared to the control group (Table 1).

To confirm the effect on platelet aggregation of both treatments, TxB2 levels were analyzed by ELISA in the serum of treated and control mice. Results showed a reduction in TxB2 levels in both ASA and Clop treated groups compared with controls, but only with Clop reached statistical significance (PBS 213.94 ± 24.52 vs. Clop 114.42 ± 17.93 pg/ml, *p* = 0.01, Figure 4E). These data suggest that Clop is the most potent anti-aggregating agent in MRL/lpr mice with LN, and point that TxB2 is a more sensitive marker to detect platelet activation compared with evidencing the presence of microthrombosis.

## Discussion

Our results confirm that MRL/lpr mice develop proliferative LN with high levels of aCL and IgG a-dsDNA antibodies (26, 27). More interestingly, acute vascular lesions are present in two thirds of the population, proving the utility of this experimental model to study the vascular pathology present in SLE with secondary APS. Our data support the idea that the underlying mechanism for microthrombosis development is related to the inflammatory response.

Macrophages are thought to be important in the pathogenesis of LN. An influx of activated macrophages can be seen in both murine and human LN (28–32), with macrophages accumulating peri- and intra-glomerularly (33, 34). In addition, pharmacological inhibition of macrophage infiltration in the kidney ameliorates SLE in mouse models (35). Our findings also evidence that macrophagic infiltration is the most consistent histological marker of inflammatory damage in MRL/lpr mice with LN and its accumulation correlates with the degree of proteinuria.

Our data suggest a relationship between the development of microthrombosis and the inflammatory response. Although we do not confirm a significant correlation among microthrombosis and degree of glomerular and extraglomerular macrophage infiltration, we do find a strong association between microthrombosis and proteinuria, and proteinuria with macrophage infiltration. Another evidence is the fact that treatment with Dexa reduced both microthrombosis and macrophage infiltration, pointing an indirect association between microthrombosis and the inflammatory response, measured as the degree of macrophage infiltration.

Other inflammatory mediators in LN are complement activated factors that act as potent soluble inflammatory anaphylotoxic and chemotactic molecules. Their stimulation promotes recruitment of neutrophils and monocytes to mediate endothelial cell activation. Some reports suggest that pathogenic aPL antibodies bind to target cells and complement (36–38), and similar mechanisms have been described in other systemic vascular disorders such as hemolytic uremic syndrome, diabetic vasculopathy, TMA, and hyperacute graft rejection (39–42). Several groups have extensively investigated the role of complement in experimental LN and APS. In fact, inhibition of C3/C5 activation complex in MRL/lpr mice led to a reduction in renal disease and a significant decrease in thrombus size compared to controls (15, 41–43). Specifically, lower renal disease and prolonged survival have been described in MRL/lpr mice by blocking C5aR, a process associated with reduced infiltration of neutrophils and macrophages (44). In agreement with this, our data demonstrate a clear association between complement activation, measured as glomerular C3 deposition, and the presence of microthrombotic lesions, suggesting that vascular lesions are associated with the inflammatory damage in MRL/lpr mice with LN. This difference is much more relevant when we analyze TMA and C3 deposition, where mice with presence of TMA have higher percentage of C3 (14.92 vs. 4.99; *p* = 0.001). This could be explained by the fact that detection of CD41 positive platelet aggregates represents an earlier phase of platelet activation, whereas the histological finding of TMA already corresponds to an established thrombotic lesion.

Our results suggest that proteinuria is a good clinical marker of renal damage in this model, being higher in mice with most severe proliferative and microthrombotic lesions. Indeed, according with data previously reported in human and mice models (18, 45, 46), proteinuria levels correlated with macrophagic infiltration.

In our study, treatment with steroids prevents proteinuria and infiltration of inflammatory cells, in agreement with previous published results (47). Surprisingly, our results demonstrate a similar effect of platelets anti-aggregation therapies on macrophagic infiltration and proteinuria, even without significantly reducing the presence of microthrombosis. Previous reports showed that the excretion of urinary TxB2, the stable thromboxane A2 hydration product, is increased in patients with LN and correlates with the activity of renal lesions and with renal function failure (48). Although the presence of thromboxane synthase has been reported in macrophages and glomerular epithelial and mesangial cells (49), we did not find any effect on TxB2 levels after Dexa treatment. In contrast, a reduction in TxB2 was observed after anti-aggregation therapies, suggesting that platelets are the main source of TxB2 in this model. Similar results have been reported in SLE patients with LN treated with ASA (47). The action of ASA depends on the irreversible inhibition of arachidonate cyclo-oxygenase activity in platelets, a process that may have an implication in macrophage/lymphocyte activation with increased production of IL-3 (50). Modulation of arachidonic acid metabolism by ASA reduces the extent of TxB2 formation, an effective inducer of platelet granule secretion and platelet aggregation (48), and our results show a trend to a lower level of TxB2 after ASA treatment. Clop acts by causing irreversible blockade of adenosine diphosphate binding to one of its receptors on the platelet surface, the P2Y12 receptor. Although the mechanisms of thrombosis may be different, previous studies point to complement activation and inflammation as common mechanisms in LN- and aPL-associated vascular pathology. Interestingly, Clop reduced TxB2 levels, demonstrating its effect as the most potent anti-aggregating agent in MRL/lpr mice with LN, not only reducing the inflammatory response, but also dropping TxB2 levels.

The observation that Clop treatment reduces aCL antibodies is somehow surprising in the absence of a primary effect of Clop on B cells. However, in addition to the presence of autoreactive B cells, the efficient production of autoantibodies is regulated by several factors, including the formation of functional germinal centers and the availability of antigen. In this respect, platelets contribute to the formation of germinal centers under certain circumstances (51). More importantly, reduction of C-reactive protein and aCL levels after treatment with ASA has been described in patients with coronary artery disease, a finding attributed to a reduction of antigen load (52).

These results point to the idea that anti-aggregation has a more potent effect than anti-inflammatory therapies, with an effect in the complement pathway and also a systemic effect by reducing sCr and aCL. Whether these changes in platelet activation promote long-term benefits in the outcome of LN remains to be defined.

One of the main limitations of the study is the severity of the disease in MRL/lpr mice. They develop a rapid and aggressive inflammatory lesion, with a high mortality rate in those older than 4 months of age, which reduces the window of intervention and prevents finding differences between treatments. Despite this, proteinuria and the degree of macrophage infiltration are very sensitive markers to the control of inflammation, which have allowed us to study the effect of anti-inflammatory and antiaggregant therapy. On the other hand, TxB2 levels appear as the most sensitive marker on platelet activation after treatment with anti-aggregants.

In summary, we have demonstrated that MRL/lpr mice with LN are a proper model to analyze the potential impact of anti-thrombotic intervention on renal inflammation. Our findings suggest that anti-aggregation treatment in combination with immunosupressors can provide additional benefits in LN. It is essential to further identify the presence of vascular lesions associated with the presence of aCL, for their implication in inflammatory damage and their association with worse evolution in renal function and in patient survival.

## Author contributions

EG-G, CG-H, and AU carried out all murine experiments (EG-G), IHC studies (EG-G, CG-H), AU and drafted the manuscript (EG-G). OT carried out histological studies at Hospital 12 de Octubre. SP-Y and DB provided the mice, collaborated in the development of the experimental model and helped to obtain the biological samples. GC participated in the design of animal experiments, statistical analysis and reviewing the results. JP and MG participated in the design of the study, performed the statistical analysis, and participated in its coordination and helped to draft the manuscript. All authors read and approved the final manuscript.

### Conflict of interest statement

The authors declare that the research was conducted in the absence of any commercial or financial relationships that could be construed as a potential conflict of interest.

## Abbreviations

aPL: antiphospholipid antibodies
APS: antiphospholipid syndrome
ASA: acetylsalicylic acid
Clop: clopidogrel
Dexa: dexamethasone
HE: hematoxylin and eosin
i.p.: intraperitoneally
IHC: immunohistochemistry
LN: lupus nephritis
PAS: Periodic acid–Schiff stain
PBS: Phosphate buffered saline
sAlb: serum albumin
sCr: serum creatinine
SLE: systemic lupus erythematosus
TMA: thrombotic microangiopathy
TF: tissue factor
TxB2: thromboxane B2.
